# Quantification of total dinutuximab concentrations in neuroblastoma patients with liquid chromatography tandem mass spectrometry

**DOI:** 10.1007/s00216-018-1198-0

**Published:** 2018-06-25

**Authors:** Mohsin El Amrani, Celina L. Szanto, C. Erik Hack, Alwin D. R. Huitema, Stefan Nierkens, Erik M. van Maarseveen

**Affiliations:** 1 Department of Clinical Pharmacy, Division of Laboratory Medicine and Pharmacy, University Medical Center Utrecht, Utrecht University, Postbus 85500, 3508 GA Utrecht, The Netherlands; 2 Laboratory of Translational Immunology, University Medical Center Utrecht, Utrecht University, P.O. Box 85500, 3508 GA Utrecht, The Netherlands; 3 Department of Pharmacy & Pharmacology, Netherlands Cancer Institute, P.O. Box 90203, 1006 BE Amsterdam, The Netherlands

**Keywords:** Dinutuximab, Ammonium sulfate sample purification, Therapeutic monoclonal antibody, Biopharmaceuticals, Quantification, Liquid chromatography tandem mass spectrometry

## Abstract

Neuroblastoma is one of the most commonly found solid tumors in children. The monoclonal antibody dinutuximab (DNX) targets the sialic acid-containing glycosphingolipid GD2 expressed on almost all neuroblastoma tumor cells and induces cell lysis. However, the expression of GD2 is not limited to tumor cells only, but is also present on central nerve tissue and peripheral nerve cells explaining dinutuximab toxicity. The most common adverse reactions are pain and discomfort, which may lead to discontinuation of the treatment. Furthermore, there is little to no data available on exposure and effect relationships of dinutuximab*.* We, therefore, developed an easy method in order to quantify dinutuximab levels in human plasma. Ammonium sulfate (AS) was used to precipitate all immunoglobulins (IgGs) in human plasma. After centrifugation, supernatant containing albumin was decanted and the precipitated IgG fraction was re-dissolved in a buffer containing 0.5% sodium dodecyl sulfate (SDS). Samples were then reduced, alkylated, and digested with trypsin. Finally, a signature peptide in complementarity determining region 1 of DNX heavy chain was quantified on LC-MS/MS using a stable isotopically labeled peptide as internal standard. AS purification efficiently removed 97.5% of the albumin fraction in the supernatant layer. The validation performed on DNX showed that within-run and between-run coefficients of variation (CV) for lower limit of quantification (LLOQ) were 5.5 and 1.4%, respectively. The overall CVs for quality control (QC) low, QC med, and QC high levels were < 5%. Linearity in the range 1–32 mg/L was excellent (*r*^2^ > 0.999). Selectivity, stability, and matrix effect were in concordance with EMA guidelines. In conclusion, a method to quantify DNX in human plasma was successfully developed. In addition, the high and robust process efficiency enabled the utilization of a stable isotopically labeled (SIL) peptide instead of SIL DNX, which was commercially unavailable.

**Figure:**
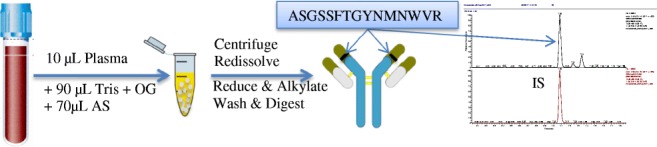
Graphical abstract

Graphical abstract

## Introduction

Neuroblastoma (NB) is the third most common childhood cancer with a prevalence of 10.2 cases per million children under the age of 15 years [1]. NB is an embryonic cancer of the post-ganglionic sympathetic nervous system which is usually formed in nerve tissues of the adrenal gland, neck, chest, or spinal cord [2]. Early diagnosis of high-risk NB is very difficult; however, depending on the stage of the disease, tumors can clearly be seen as a lump under the skin. Treatment strategies of NB is dependent on the risk group it has been categorized to, since some cases of NB can show spontaneous and complete regression [1, 3–5]. However, the long-term survival of high-risk NB is between 35 to 45% despite multimodal treatment [6–8]. Therefore, a new treatment strategy based on the chimeric, mouse-human, monoclonal antibody dinutuximab (CH14.18/SP2/0; Unituxin™; DNX) has been developed to target and eradicate the remaining NB cells in order to prevent relapse [9, 10]. DNX was approved by European Medicines Agency (EMA) and Food and Drug Administration (FDA) in 2015 and is used in combination with granulocyte-macrophage colony-stimulating factor, interleukin-2, and isotretinoin [10, 11]. This therapeutic antibody targets the sialic acid-containing glycosphingolipid GD2 which is overexpressed on almost all NB tumor cells and induces cell lysis trough complement-dependent cytotoxicity and cell necrosis and apoptosis trough antibody-dependent cell-mediated cytotoxicity [8, 10, 12, 13]. However, treatment with DNX causes neuropathic pain due to GD2 presence on nerve cells; this necessitates the use of opioids prior to, during, and for 2 h after administration of DNX in order to manage pain [10]. A method to quantify DNX levels in plasma can potentially lead to new insights for personalize dosing to increase efficacy and reduce toxicity. Furthermore, it has been estimated that up to 37% of patients develop anti-drug antibodies (ADA) which could have a profound impact on drug clearance [14–16], and thus therapeutic drug monitoring of these patients is of great value.

To date, three ligand binding assays are described, two based on anti-idiotypic antibodies to DNX and the other is a cell-based enzyme-linked immunosorbent assay (ELISA) using GD2 expressing melanoma cell line [17–19]. However, the generation of these cell lines and antibodies requires specific skills and facilities. Recently, the introduction of highly sensitive liquid chromatography tandem mass spectrometry (LC-MS/MS) technology has enabled monoclonal antibody quantification. LC-MS/MS possesses notable advantages over ELISA methods such as faster assay setup times, wider linear dynamic rage, and most importantly higher selectivity [20, 21]. Therefore, we have developed an easy method to quantify total DNX in plasma using a novel sample preparation in combination with tandem mass spectrometry analysis.

## Materials and methods

### Chemicals and reagents

Dinutuximab (CH14.18/SP2/0; Unituxin®; DNX) was obtained from United Therapeutics Europe, Ltd. (Chertsey, United Kingdom) as a solution of 3.5 mg/mL. Dinutuximab beta (CH14.18/CHO; Isquette®; DNX-β) was obtained from Retschler Biotechnologie GmbH (Laupheim, Germany) as a solution of 4.5 mg/mL. As internal standard (IS) stable isotopically labeled (SIL) peptide “ASGSSFTGYNMNWV(R 13C_6_,15N_4_)” was obtained from Pepscan Presto BV (Lelystad, The Netherlands). TPCK-Trypsin was supplied by Thermo Scientific as a lyophilized powder and was dissolved in acetic acid (50 mM) to a concentration of 10 μg/μL, aliquoted in Eppendorf LoBind Microcentrifuge tubes and stored at − 80 °C. All other chemicals, reagents, and LC-MS grade mobile phase solvents were obtained from Sigma-Aldrich (Saint Louis, MO).

### Preparation of standards, internal standard, and quality control samples

The working DNX standard solution (32 μg/mL) was prepared by pipetting 30 μL stock solution Unituxin (3.5 μg/μL) and 180 μL pooled plasma in a LoBind Eppendorf tube to obtain a concentration of 500 μg/mL. This solution was further diluted to 32 μg/mL in pooled plasma and aliquots were stored at − 80 °C. Standard concentrations of 1, 2, 4, 8, 16, and 32 μg/mL were freshly prepared from the working standard solution by serial dilution in pooled plasma. The working internal standard SIL peptide solution “ASGSSFTGYNMNWV(R 13C_6_,15N_4_)” at a concentration of 0.5 mg/L was prepared in tris(hydroxymethyl)aminomethane (Tris) buffer pH 8.5, 100 mM containing 0.5% octyl glucoside (OG). Quality control samples (QCs) at lower limit of quantification (LLOQ) (1 μg/mL), QC low (3 μg/mL), QC med (15 μg/mL), and QC high (25 μg/mL) were prepared in pooled plasma from a separate batch. Aliquots were stored at − 80 °C.

### Instrumentation and chromatographic conditions

Sample reduction, alkylation, and digestion was performed on Eppendorf™ ThermoMixer C. All measurements were performed on an Ultimate 3000 UHPLC Dionex (Sunnyvale, CA) coupled to a TSQ Quantiva, Thermo Fisher (Waltham, MA). The analytical column was Acclaim™, RSLC 120, C18, 2.1 × 100 mm, 2.2 μm particle size, Thermo Fisher (Waltham, MA), The Guard column was the SecurityGuard column ULTRA C18, 2.1 mm (Phenomenex) and were maintained at 50 °C. The mobile phases were (A) 0.1% formic acid in water, and (B) 0.1% formic acid in acetonitrile. The LC gradients in minutes per percentage of mobile phase B were 0.0 (min)/2 (% B), 7.5/24, 7.6/85, 8.5/85, 8.6/2, and 10.5/2. The flow rate was 0.6 mL/min and the run time was 10.5 min. The mass spectrometer was operated in positive mode with spray voltage of 3.5 kV, ion transfer tube temperature 350 °C, vaporizer temperature 300 °C, aux gas pressure 15 Arb, sheath gas pressure 50 Arb, collision energy 30 V, collision gas pressure 2.5 mTorr, and radio frequency (RF) lens 110 V. The precursor ions and product ions settings are listed in Table 1 for DNX and for the SIL internal standard.

**Table 1:** Mass spectrometer conditions for selected reaction monitoring (SRM) transitions for the signature peptide (liberated from DNX after digestion with trypsin) and the SIL internal standard

Peptide sequence	Used as	Precursor (*m*/*z*)	Product (*m*/*z*)	Product ion type
ASGSSFTGYNMNWVR	Qualifier	838.88	819.39	Y6
ASGSSFTGYNMNWVR	Quantifier	838.88	1039.47	Y8
ASGSSFTGYNM(O)NWVR	Oxidation check	846.88	1055.47	Y8
ASGSSFTGYNMNWVR	Qualifier	838.88	1140.52	Y9
ASGSSFTGYNMNWVR [13C6,15 N4]	Qualifier	843.90	829.39	Y6
ASGSSFTGYNMNWVR [13C6,15 N4]	Quantifier	843.90	1049.47	Y8
ASGSSFTGYNM(O)NWVR [13C6,15 N4]	Oxidation check	851.90	1065.47	Y8
ASGSSFTGYNMNWVR [13C6,15 N4]	Qualifier	843.90	1150.52	Y9

### Sample preparation for LC-MS/MS analysis

**Fig. 1:**
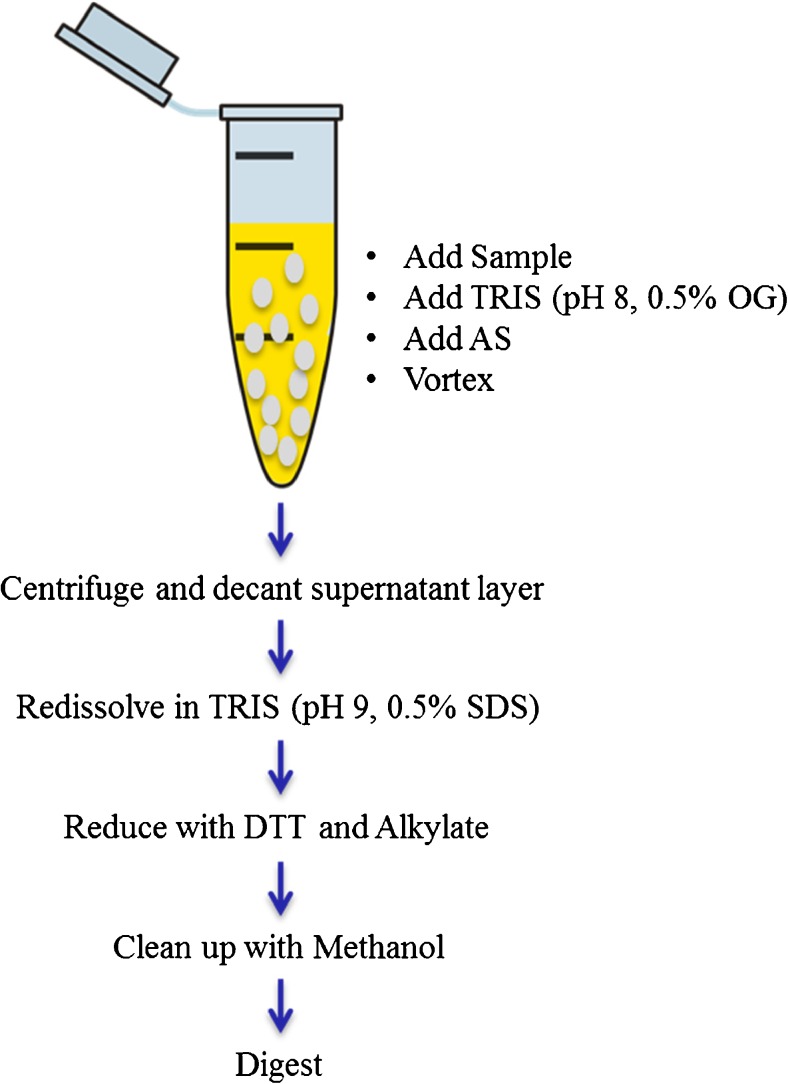
Sample purification workflow using AS

Ammonium sulfate (AS) protein precipitation method was chosen because of its inherent simplicity, high sample throughput, and fast work flow (Fig. 1). In brief, 10 μL (sample, standard, or QC) was diluted with 90 μL Tris (50 mM, pH 8, 0.5% OG) in 1 mL LoBind 96-well plate. Then, 70 μL AS (saturated) was added to each sample followed by 1 min mixing at room temperature. The 96-well plate was centrifuged at 4000 G for 5 min to collect the IgG pellet at the bottom. The supernatant containing albumin was decanted and the pellet was re-dissolved in 50 μL Tris (100 mM, pH 9, 0.5% sodium dodecyl sulfate (SDS), 20 mM 1,4-dithiothreitol (DTT)). Then, the well plate was placed in a ThermoMixer at 60 °C, 1000 rpm for 30 min to reduce the disulfide bonds. Samples were alkylated by adding 20 μL iodoacetamide (IAA) (100 mM dissolved in ultrapure water) and placed on a ThermoMixer at 37 °C for 30 min in the dark. Then, 150 μL ultrapure water was added and mixed for 1 min to dilute the SDS and IAA. After mixing, methanol was added to precipitate the IgG fragments and the well plate was centrifuged at 4000 G for 5 min. The supernatant containing SDS and IAA was decanted. Then, 90 μL IS working solution was added, followed by 10 μL Trypsin (2 μg/μL) and the samples were placed on the ThermoMixer for overnight digestion at 37 °C, 800 rpm. Trypsin activity was stopped by adding 30 μL 10% formic acid, 5% trifluoroacetic acid (TFA) in acetonitrile. Finally, 25 μL was injected on an LC-MS/MS.

### Signature peptide selection

Tryptic digestion is regularly used to obtain peptides to enable low quantification levels of therapeutic monoclonal antibodies (mAb) in plasma with a triple quadrupole mass spectrometer. DNX sequence was obtained from the international immunogenetics information system® (http://imgt.org). After in silico digesting the variable chains with the online tool from institute of systems biology (http://db.systemsbiology.net), potential signature peptide candidates having amino acids in the range of 6 < *n* < 20 were identified. These amino acids were then screened using pBlast (https://blast.ncbi.nlm.nih.gov/Blast.cgi?PAGE=Proteins). Finally, the retention time (RT) of the surrogate peptide candidates were identified on the Q Exactive (Thermo Scientific) and the signal intensity of all peptides were compared.

### Albumin determination

After sample purification with AS, the remaining concentration albumin in the pellet was measure by means of immunonephelometry on the BN ProSpec® System (SIEMENS).

In brief, 200 μL plasma from a healthy donor was diluted with 1800 μL Tris (50 mM, pH 8, 0.5% OG) in a test tube in triplicates. Then, 1400 μL AS (saturated) was added to each tube followed by 1 min mixing at room temperature. Then the test tubes were centrifuged at 4000 G for 5 min to collect the IgG pellet at the bottom. The supernatant was decanted to the waste and the pellet was re-dissolved in 200 μL phosphate-buffered saline (PBS). The remaining albumin in the pellet and in the original plasma sample was measured on the BN ProSpec.

### AS concentration and DNX recovery

Ten microliters of standard 6 (32 mg/L) was pipetted 12 times in lobind 96-deep well plate followed by 90 μL Tris (pH 8, 0.5% OG). Then, 50, 60, 70, and 80 μL saturated AS was added in triplicates followed by mixing and centrifugation. Samples were then prepared and analyzed according to chapter 2.4.

### Methanol concentration and DNX recovery

Ten microliters of standard 6 (32 mg/L) was pipetted 18 times in lobind 96-deep well plate followed by 90 μL Tris (pH 8, 0.5% OG). Samples were then prepared according to chapter 2.4 with the following modifications; following alkylation 0, 50, 100, 150, 200, 250 μL water was added in triplicates followed by 550, 500, 450, 400, 350, 300 μL methanol also in triplicates.

### Comparison between DNX and DNX-β

A test was performed to determine whether concentrations of DNX and DNX-β were similar. A DNX-β control sample was prepared at a concentration of 15 mg/L and was run in duplicate against DNX calibration curve. DNX standards and the DNX-β control sample were prepared as described in chapter 2.2.

### Patient infusion samples

After obtaining parental informed consent, eight peripheral blood samples of 2 mL (in EDTA-treated tubed) were drawn from one pediatric patient each time before and after DNX infusion. The patient was given a daily infusion of 14.01 mg DNX for 10–20 h during a 4-day treatment period. Peripheral blood samples were centrifuged at 600 G for 15 min. Following centrifugation, the supernatant (plasma) was carefully removed from the cell pellet. Plasma was aliquoted into 1–2 volumes of 0.5 mL and immediately stored at − 80 °C. After storage, samples were analyzed in duplicates according to the procedure described in chapter 2.4.

### Validation

The method was validated according to EMA guidelines which dictate the investigation of parameters such as, LLOQ, linearity, selectivity, matrix effect, carry-over, auto sampler stability, freeze/thaw stability, within-run and between-run precision and accuracy [22]. LLOQ was chosen based on the reference ELISA used to generate pharmacokinetic data for FDA approval [23] and was determined by comparing the signal obtained from standard 1 (1 mg/L) against a pooled normal human plasma after sample preparation according to the above described method. The acceptance criterion is that the signal of standard 1 (LLOQ) should be at least five times higher than the signal obtained for the pooled normal human plasma at the RT of the signature peptide. The linear dynamic range of the standard curve was established based on theoretical peak levels that would be obtained in patients and was investigated by measuring six standards at concentrations 1.00, 2.00, 4.00, 8.00, 16.00, and 32.00 μg/mL for 3 days. The acceptance criterion for the back calculated concentration for LLOQ was 20% of the nominal value and for the remaining standard 15% of the nominal value. Selectivity was tested by evaluating ten human plasma samples from healthy donors and comparing the MS signal intensity at the RT of the signature peptide against LLOQ signal. The noise signal intensity obtained for the blank plasma samples at the RT of DNX signature peptide should be less than 20% of the LLOQ signal. Matrix effect was tested by spiking human plasma samples from healthy donors at QC low (3 mg/L) and QC high (25 mg/L) and determining the concentration with the calibration curve. The back calculated concentration should be within 15% of the nominal value. Carry-over was tested by injecting digested pooled normal human plasma sample after a high standard. The acceptance criterion, in this case, was a signal obtained at the RT of DNX signature peptide of less than 20% of the LLOQ and a signal obtained for the IS under 5%. Auto sampler stability was tested by re-injecting the validation samples the next day and comparing the results to those of the day before. Freeze and thaw stability was validated by analyzing a QC low and QC high sample in fivefold during 3 days at which samples were thawed and tested, and the remaining samples were stored again at − 80 °C, and subjected to the same procedure next day. Within-run and between-run precision and accuracy was evaluated during 3 days by analyzing LLOQ (1 μg/mL), QC low (3 μg/mL), QC med (15 μg/mL), and QC high (25 μg/mL) in fivefold each day. The data obtained for each concentration was evaluated with single factor ANOVA. Accuracy was expressed as percentage bias. Within-run and between-run precision was expressed as percent coefficient of variation (% CV) and was calculated by taking the squared root of the mean squares (MS) and dividing this by the overall mean concentration times 100%.

## Results and discussion

### Method development

A novel sample preparation method was developed based an optimized combination of established methods from literature [20, 24–27]. AS purification was found to be a fast, easy, and efficient way to remove plasma albumin which comprises of approximately 60% of total plasma proteins. Furthermore, the protein pellet could easily be re-dissolved in working buffers, suggesting that AS did not denature the IgGs and kept the tertiary structure intact. The use of a MS compatible non-ionic surfactant, octyl glucoside (OG), with AS aided in the removal of phospholipids which are notorious MS-ionization suppressants. SDS is a widely used inexpensive ionic detergent, and is very efficient in protein unfolding and solubilization when used under reducing conditions in the presence of DTT. However, SDS is not compatible with trypsin nor with MS thus removal prior to these steps is essential. Using our method, SDS was efficiently removed by protein precipitation with methanol, as SDS remains in solution in the aqueous layer. This was verified by evaporating the supernatant layer under nitrogen. Upon the addition of water, foam was clearly visible after agitating the test tube. Methanol precipitation also allowed for efficient removal of the remaining salts. The internal standard was a SIL peptide ASGSSFTGYNMNWV(R 13C_6_,15N_4_) and was introduced to the samples prior to digestion. The internal standard allowed for correction of ionization suppression and injection volume differences during MS analysis. Importantly, digestion with trypsin needed to be reproducible between different patients because the SIL peptide cannot correct for digestion efficiency. Here, we found that the protein pellet was completely dissolved in all patients containing different levels of IgG’s during matrix effect studies.

### Signature peptide selection

In-silico digestion of the variable light (VL) and heavy chain (VH) provided 10 potential candidates that were between 6 and 20 amino acids long and that could possibly be used as signature peptide (Table 2). After preforming a protein blast search, three peptide candidates (VL1, VL19, and VL67) were found to be endogenous to humans and were dismissed. VL 33 was also dismissed because it contained asparagine followed by glycine. Glycine, a small amino acid group, is not capable of shielding asparagine from deamination reaction. The remaining candidates were screened using high-resolution mass spectrometry after tryptic digestion (data not shown). Signals for peptide VH68 and VL60 were found to be low, probably due to charge interference caused by the aspartic acid group (D) near the trypsin digestion sites (K and R). Peptide VH24 (ASGSSFTGYNMNWVR) and VH44 (SLEWIGAIDPYYGGTSYNQK) from the complementarity determining region (CDR) 1 and CDR 2, respectively, were found to have the highest signal intensity and both were optimized for collision energy and RF lens settings on the triple quadrupole. After optimizing the digestion condition, the signal intensity of VH44 was found to be too low to allow for quantification at the required LLOQ of 1 μg/mL. Therefore, VH44 peptide was omitted and the validation was performed on the VH24 peptide. Although VH24 peptide contains a methionine group, no oxidation peaks were found after overnight digestion (data not shown).

**Table 2:** Peptides with amino acids (6 < *n* < 20) obtained after in-silico digestion of the variable chains. Results for query cover and identification percentages were obtained from pBlast using human library from Swiss-Prot database

Location	Sequence	Mass	Query cover (%)	Identification (%)
VH24	ASGSSFTGYNMNWVR	1676.7485	100	73
VH44	SLEWIGAIDPYYGGTSYNQK	2262.0713	80	63
VH68	ATLTVDK	747.4247	100	86
VH75	SSSTAYMHLK	1124.5405	100	88
VL1	EIVMTQSPATLSVSPGER	1901.9637	100	100
VL19	ATLSCR	650.3290	100	100
VL25	SSQSLVHR	913.4850	75	100
VL33	NGNTYLHWYLQKPGQSPK	2131.0719	94	82
VL60	FSGVPDR	777.3889	85	100
VL67	FSGSGSGTDFTLK	1303.6164	100	100

### Level of albumin remaining after AS purification

**Fig. 2:**
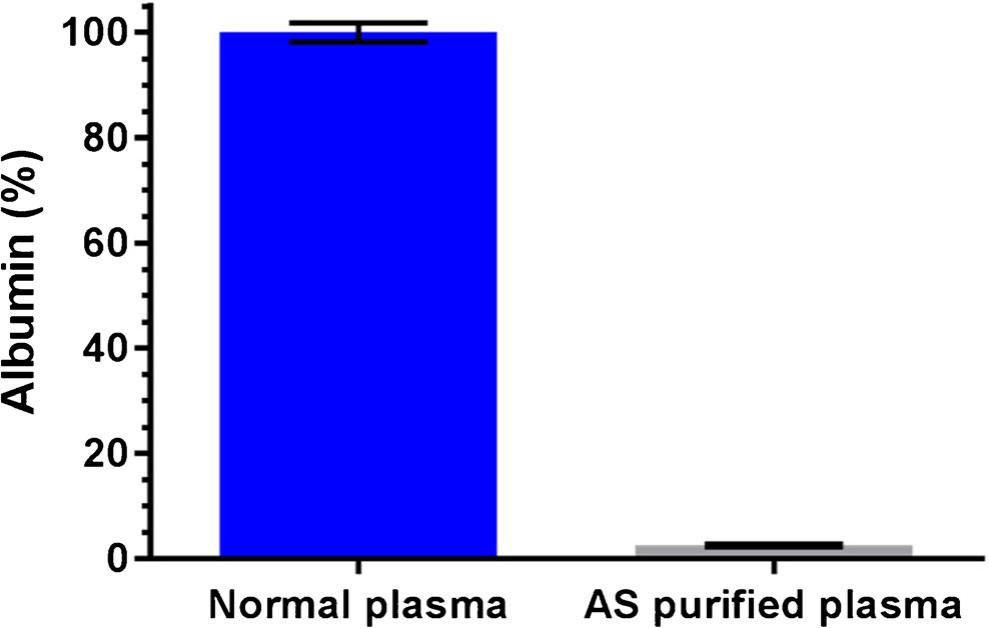
Level of albumin remaining without pretreatment (normal plasma) and with AS pretreatment (AS purified plasma), error bars represent SD with *n* = 3

The albumin concentration of the untreated plasma sample and the AS pellet re-dissolved in PBS buffer were measured in triplicate by means of immunonephelometry. The untreated plasma sample had a mean concentration albumin of 39.5 g/L with a standard deviation (SD) of 0.72 g/L and the AS pretreated plasma sample had a mean remaining albumin concentration of 0.98 g/L with an SD of 0.1 g/L. This translates to a highly efficient depletion of 97.5% albumin with AS pretreatment (Fig. 2).

### AS concentration and DNX recovery

This test was performed to determine the concentration AS needed that provides the highest recovery. Increasing concentrations AS were tested starting from 33.3% going up to 44.4%.

**Fig. 3:**
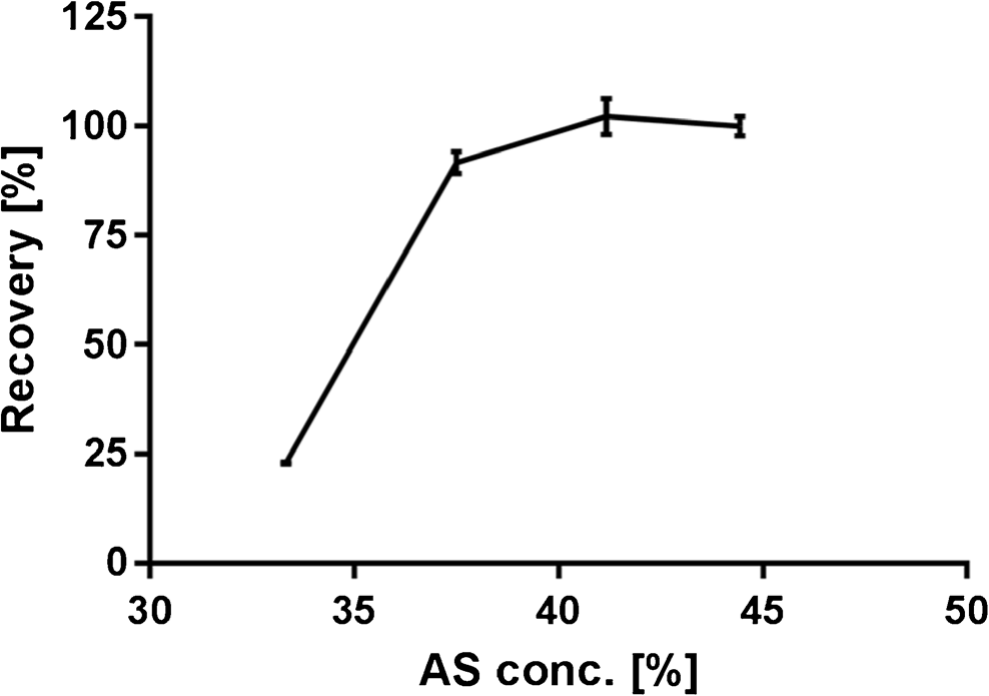
Concentration AS plotted against DNX recovery, error bars represent SD with *n* = 3

From the results obtained, we see that at 37.5% AS the line started to bend reaching a plateau at an AS concentration of 41.2% (Fig. 3). This translates to 70 μL saturated AS per 100 μL solution consisting of 10 μL sample and 90 μL Tris (50 mM, pH 8, 0.5% OG).

### Methanol concentration and DNX recovery

**Fig. 4:**
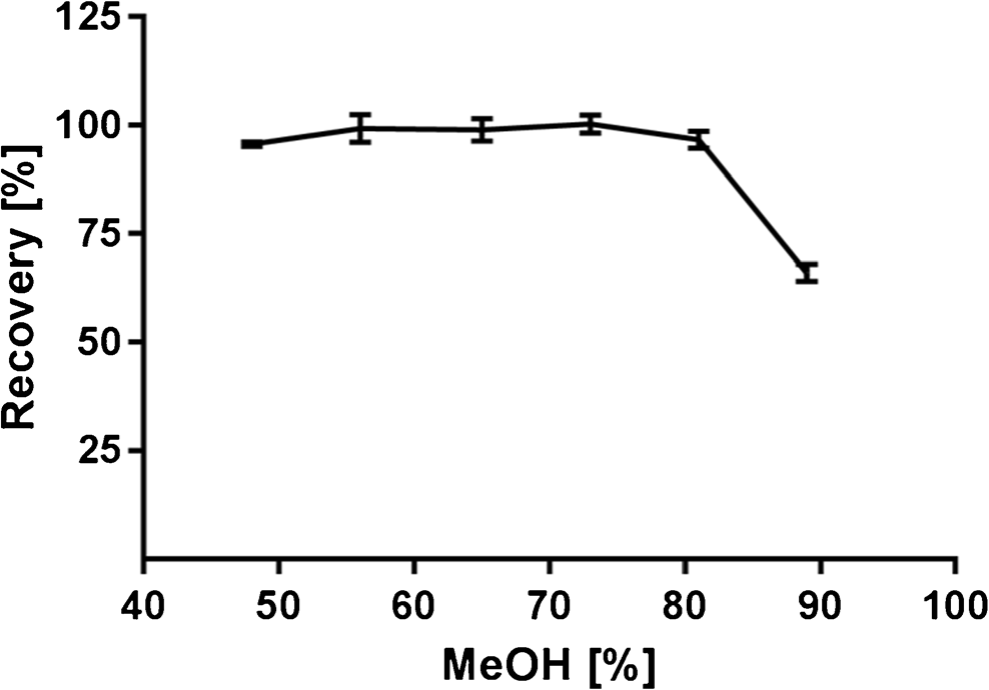
Methanol concentration plotted against DNX recovery, error bars represent SD with *n* = 3

The second step in the procedure that could lead to loss of DNX is the water–methanol washing step. Water was used to dilute the SDS and methanol was used to precipitate the proteins in order to obtain a pellet after centrifugation. From the obtained results, we see that the highest concentration methanol tested resulted in the lowest recovery (Fig. 4). This is counterintuitive since we expect the opposite. In fact, the reason for the signal drop is not due to loss of DNX in the washing step, but rather due to ionization suppression or trypsin denaturation caused by inefficient removal of SDS. Here, we notice that we need to introduce at least 50 μL of water per sample to dilute and remove SDS in the supernatant layer. Using 48% methanol, we noticed a decrease in DNX recovery and therefore, we chose 65% methanol as the optimum concentration which lies in the middle of the plateau. This corresponds to 400 μL methanol per 70 μL sample diluted with 150 μL water.

### Comparison between DNX and DNX-β

DNX-β (CH14.18/CHO; Dinutuximab beta; Isquette) received marketing approval in 2017 and is currently used to treat new patients suffering from NB. DNX (CH14.18/SP2/0; Unituxin) was withdrawn from the market in March 2017. However, patients on Unituxin will continue to receive this formulation. DNX-β can be given without co-administration of interleukin-2, which induces inflammatory side effects [28]. These two drugs contain the same peptide sequences, only glycosylation differences can occur due to the different cell lines used. Since measurements are based on signal obtained from the signature peptide, these two drugs were found to be well correlated with a bias of 8.8% and RSD of 1.13%. The latter illustrates one of the advantages of antibody quantification using LCMSMS.

### Patient infusion data

**Fig. 5:**
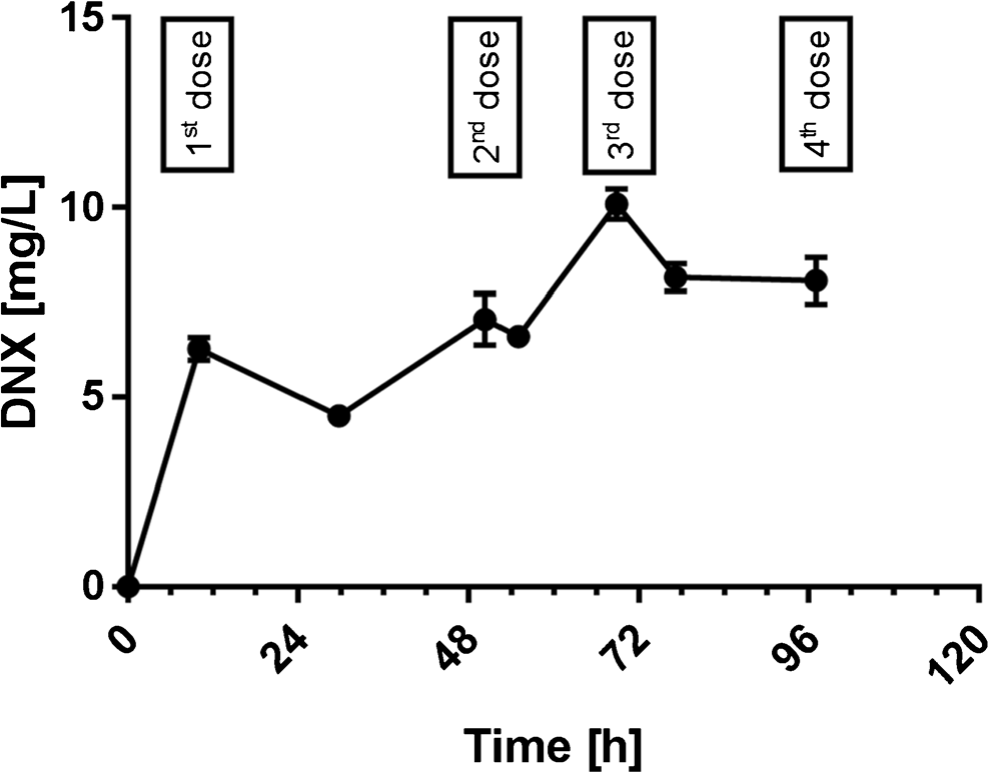
DNX concentration data obtained from one patient dosed during 4 days, each time with a 10–20 h infusion of 14.01 mg DNX, error bars represent SD with *n* = 2

One patient was monitored for DNX concentration with the above described method during the 4-day treatment period. Samples were drawn before infusion and at the end of a 10 to 20 h DNX infusion. The third dose showed the highest DNX concentration (Fig. 5). This was probably due to relative short resting period (8 h) in between dose 2 and 3. The resting period in between dose 1 and 2 and between dose 3 and 4 were 30 and 18 h respectively.

### Validation

**Fig. 6:**
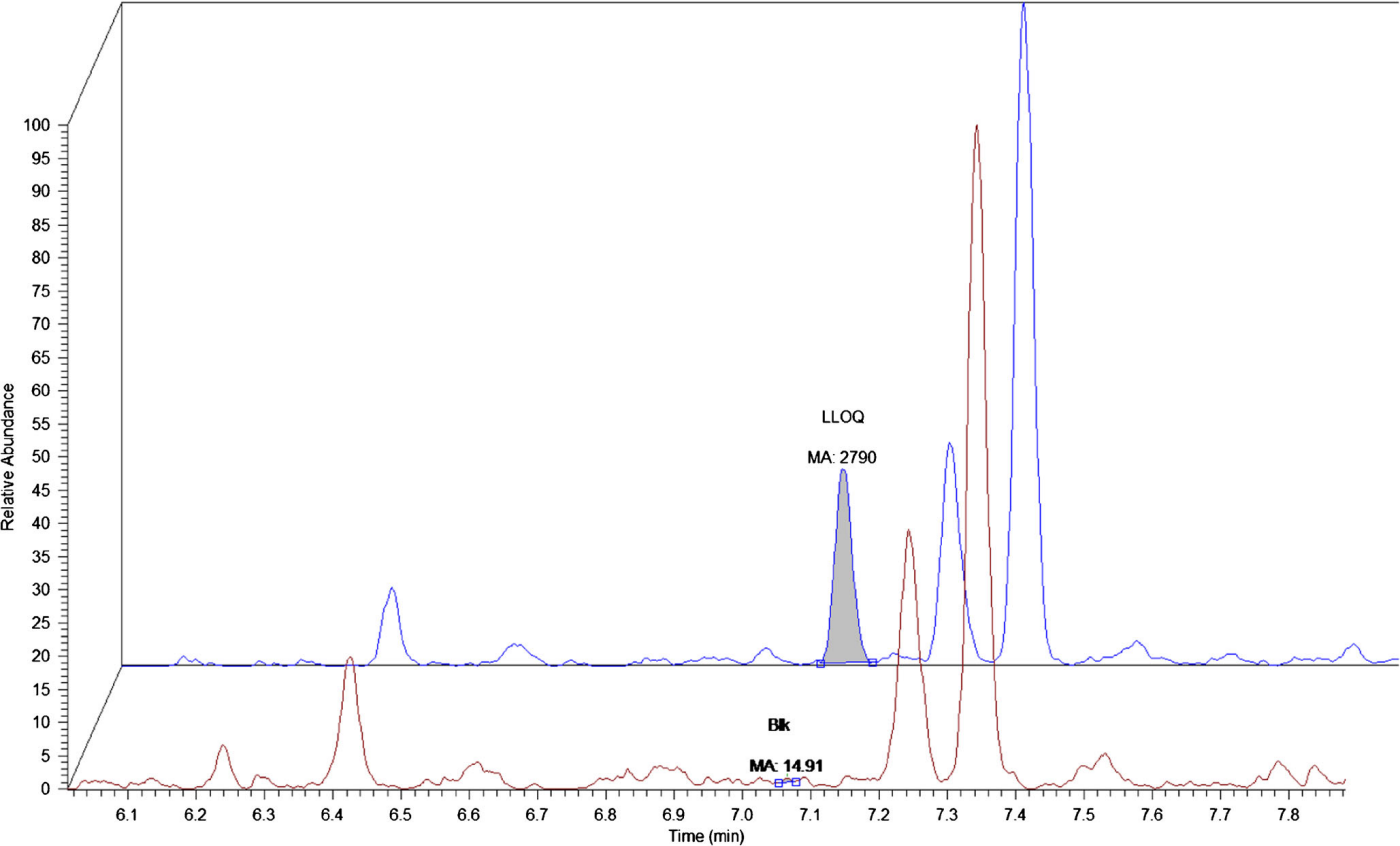
LLOQ at 1 μg/mL tested against a blank pooled normal human plasma sample

**Fig. 7:**
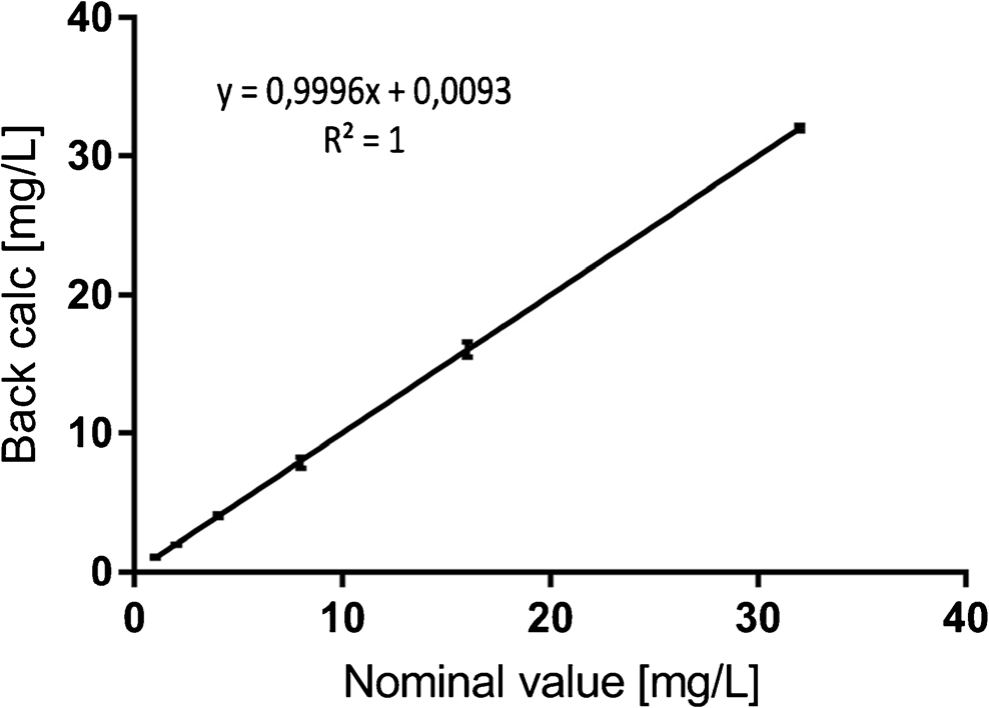
Linearity test, standard curve with SD error bars at each standard level tested during 3 days

The LLOQ was first determined by analyzing DNX spiked in pooled normal human plasma sample at a concentration of 1 μg/mL (Fig. 6). The signal-to-noise ratio (S/N) was found to be approximately 187, which is well above the EMA guidelines (threshold of S/*N* > 5). Linearity was evaluated during 3 days using the following standards; 1.00, 2.00, 4.00, 8.00, 16.00, 32.00 μg/mL. The back calculated concentrations were found to be in agreement with guidelines with a determination coefficient *R*^2^ > 0.999 (Fig. 7). Selectivity and carry-over were evaluated by analyzing ten normal human plasma samples and measuring the signal intensity at the RT of the signature peptide in relation to LLOQ signal intensity. Table 3 lists the percentage signal in relation to LLOQ signal for DNX signature peptide and for the SIL IS. Matrix effect was tested by spiking DNX at QC low and QC high levels in seven different human plasma samples. The average bias was in concordance with EMA guidelines of 15% (Table 4). Auto sampler stability was tested by re-injecting the validation samples after 24 h. Signal intensities and concentration values were found to be in agreement with previous run (data not show). Freeze and thaw stability was tested during 3 days with overall CV of around 4% for both QC low and QC high and a bias of 1.4 and 5%, respectively. And finally, within-run and between-run precision and accuracy for LLOQ, QC low, QC med, and QC high were evaluated during 3 days in fivefold and were found to be well within the acceptance criteria with CV in the range of 5% which is three times lower than the set limit see Table 5.

**Table 3:** Selectivity and carry over test with randomly chosen blank human plasma samples

Blank human plasma sample	% Signal in relation to LLOQ signal	% Signal in relation to IS signal
Plasma pool − IS (carry-over)	12	0.16
Plasma pool + IS	3	112.05
Sample 1	2	0.08
Sample 2	5	0.06
Sample 3	11	0.02
Sample 4	8	0.04
Sample 5	5	0.06
Sample 6	7	0.03
Sample 7	1	0.07
Sample 8	1	0.05
Sample 9	5	0.00
Sample 10	8	0.03

**Table 4:** Matrix effect test, seven human plasma samples spiked at QC low (3 mg/L) and QC high level (25 mg/L)

Sample nr	Measured [mg/L]	Bias	Measured [mg/L]	Bias [%]
1	2.80	− 6.7	24.42	− 2.3
2	2.60	− 13.3	25.98	3.9
3	3.16	5.2	27.61	10.4
4	3.29	9.5	26.28	5.1
5	3.26	8.6	29.00	16.0
6	3.24	8.2	26.82	7.3
7	3.14	4.7	26.87	7.5

**Table 5:** Accuracy and precision validation data for QC’s at LLOQ, low, medium, and high levels. Within-run data were based on five replicates and between-run data on three different days

	Precision (% CV)			Accuracy (% bias)
QC	Within-run	Between-run	Overall	Overall
LLOQ	5.5	1.4	5.7	6.4
Low	4.4	1.2	4.6	0.2
Med	2.9	2.0	3.5	2.9
High	2.9	3.4	4.5	4.6

## Discussion and conclusion

A novel sample work-up method utilizing AS in combination with SDS was developed for the quantification of total DNX in human plasma. The AS precipitation method facilitated efficient removal of albumin while retaining a high recovery for DNX. The development of a DNX LC-MS/MS method was performed because of the clinical need for reliable method to quantify DNX in plasma for ongoing pharmacokinetic studies and future drug monitoring. This method measures total (free and bound) DNX concentration in plasma while ELISA methods measure free DNX concentrations. Previous studies in animals and in humans showed that total mAb fraction measured by LC-MS/MS provide similar PK profiles as those obtained by ELISA’s free mAb fraction [29–33]. Furthermore, Willrich and colleagues have shown that total infliximab was strongly correlated to free infliximab even in a subset of samples containing anti-drug antibodies [26]. These data show that similar results can be obtained with assays that are fundamentally different. In addition, LC-MS/MS methods are in many ways analytically superior to ligand binding assays due to their high specificity, wider linear dynamic range, and higher accuracy and precision.

The method was validated in concordance with the latest EMA/FDA guidelines. Furthermore, excellent validation data were obtained. This was mainly due to the ease of use of the method and to the efficient and robust digestion process which was achieved by incorporating SDS in the sample work-up. Finally, the here described method can be used as a template for the quantification other therapeutic monoclonal antibodies in plasma.

## References

[1] Maris JM (2010). Recent advances in neuroblastoma. N Engl J Med.

[2] Hara J (2012). Development of treatment strategies for advanced neuroblastoma. Int J Clin Oncol.

[3] Tsubota S, Kadomatsu K (2017). Origin and mechanism of neuroblastoma. Oncoscience.

[4] Carlsen NL (1990). How frequent is spontaneous remission of neuroblastomas? Implications for screening. Br J Cancer.

[5] Yamamoto K, Hanada R, Kikuchi A, Ichikawa M, Aihara T, Oguma E, Moritani T, Shimanuki Y, Tanimura M, Hayashi Y (1998). Spontaneous regression of localized neuroblastoma detected by mass screening. J Clin Oncol.

[6] Matthay KK, Reynolds CP, Seeger RC, Shimada H, Adkins ES, Haas-Kogan D, Gerbing RB, London WB, Villablanca JG (2009). Long-term results for children with high-risk neuroblastoma treated on a randomized trial of myeloablative therapy followed by 13-cis-retinoic acid: a children's oncology group study. J Clin Oncol.

[7] Pinto NR, Applebaum MA, Volchenboum SL, Matthay KK, London WB, Ambros PF, Nakagawara A, Berthold F, Schleiermacher G, Park JR, Valteau-Couanet D, Pearson AD, Cohn SL (2015). Advances in risk classification and treatment strategies for neuroblastoma. J Clin Oncol.

[8] Amoroso L, Haupt R, Garaventa A, Ponzoni M. Investigational drugs in phase II clinical trials for the treatment of neuroblastoma. Expert Opin Investig Drugs. 2017, 1–13. doi: 10.1080/13543784.2017.1380625. 10.1080/13543784.2017.1380625 28906153

[9] Castel V, Segura V, Canete A (2010). Treatment of high-risk neuroblastoma with anti-GD2 antibodies. Clin Transl Oncol.

[10] Dhillon S (2015). Dinutuximab: first global approval. Drugs.

[11] Ploessl C, Pan A, Maples KT, Lowe DK (2016). Dinutuximab: an anti-GD2 monoclonal antibody for high-risk neuroblastoma. Ann Pharmacother.

[12] McGinty L, Kolesar J (2017). Dinutuximab for maintenance therapy in pediatric neuroblastoma. Am J Health Syst Pharm.

[13] Barker E, Mueller BM, Handgretinger R, Herter M, Yu AL, Reisfeld RA (1991). Effect of a chimeric anti-ganglioside GD2 antibody on cell-mediated lysis of human neuroblastoma cells. Cancer Res.

[14] Ozkaynak MF, Sondel PM, Krailo MD, Gan J, Javorsky B, Reisfeld RA, Matthay KK, Reaman GH, Seeger RC (2000). Phase I study of chimeric human/murine anti-ganglioside G(D2) monoclonal antibody (ch14.18) with granulocyte-macrophage colony-stimulating factor in children with neuroblastoma immediately after hematopoietic stem-cell transplantation: a Children's Cancer group study. J Clin Oncol.

[15] Gilman AL, Ozkaynak MF, Matthay KK, Krailo M, Yu AL, Gan J, Sternberg A, Hank JA, Seeger R, Reaman GH, Sondel PM (2009). Phase I study of ch14.18 with granulocyte-macrophage colony-stimulating factor and Interleukin-2 in children with neuroblastoma after autologous bone marrow transplantation or stem-cell rescue: a report from the Children's Oncology Group. J Clin Oncol.

[16] Handgretinger R, Baader P, Dopfer R, Klingebiel T, Reuland P, Treuner J, Reisfeld RA, Niethammer D (1992). A phase I study of neuroblastoma with the anti-ganglioside GD2 antibody 14.G2a. Cancer Immunol Immunother.

[17] Siebert N, Seidel D, Eger C, Brackrock D, Reker D, Schmidt M, Lode HN (2013). Validated detection of anti-GD2 antibody ch14.18/CHO in serum of neuroblastoma patients using anti-idiotype antibody ganglidiomab. J Immunol Methods.

[18] Soman G, Yang X, Jiang H, Giardina S, Mitra G (2011). Comparison of GD2 binding capture ELISA assays for anti-GD2-antibodies using GD2-coated plates and a GD2-expressing cell-based ELISA. J Immunol Methods.

[19] Desai AV, Fox E, Smith LM, Lim AP, Maris JM, Balis FM (2014). Pharmacokinetics of the chimeric anti-GD2 antibody, ch14.18, in children with high-risk neuroblastoma. Cancer Chemother Pharmacol.

[20] El Amrani M, van den Broek MP, Gobel C, van Maarseveen EM (2016). Quantification of active infliximab in human serum with liquid chromatography-tandem mass spectrometry using a tumor necrosis factor alpha -based pre-analytical sample purification and a stable isotopic labeled infliximab bio-similar as internal standard: a target-based, sensitive and cost-effective method. J Chromatogr A.

[21] Ladwig PM, Barnidge DR, Willrich MAV. Mass spectrometry approaches for identification and quantitation of therapeutic monoclonal antibodies in the clinical laboratory. Clin Vaccine Immunol. 2017, 24(5). doi: 10.1128/CVI.00545-16. 10.1128/CVI.00545-16 PMC5424237 28274937

[22] Agency EM (2011) Guideline on bioanalytical method validation. EMA. http://www.ema.europa.eu/docs/en_GB/document_library/Scientific_guideline/2011/08/WC500109686.pdf. Accessed 16-October 2017.

[23] FDA (2014) Clinical Pharmacology and Biopharmaceutics Review. https://www.accessdata.fda.gov/drugsatfda_docs/nda/2015/125516Orig1s000ClinPharmR.pdf. 2018.

[24] An B, Zhang M, Johnson RW, Qu J (2015). Surfactant-aided precipitation/on-pellet-digestion (SOD) procedure provides robust and rapid sample preparation for reproducible, accurate and sensitive LC/MS quantification of therapeutic protein in plasma and tissues. Anal Chem.

[25] Zhou JY, Dann GP, Shi T, Wang L, Gao X, Su D, Nicora CD, Shukla AK, Moore RJ, Liu T, Camp DG, Smith RD, Qian WJ (2012). Simple sodium dodecyl sulfate-assisted sample preparation method for LC-MS-based proteomics applications. Anal Chem.

[26] Willrich MA, Murray DL, Barnidge DR, Ladwig PM, Snyder MR (2015). Quantitation of infliximab using clonotypic peptides and selective reaction monitoring by LC-MS/MS. Int Immunopharmacol.

[27] Chang YH, Gregorich ZR, Chen AJ, Hwang L, Guner H, Yu D, Zhang J, Ge Y (2015). New mass-spectrometry-compatible degradable surfactant for tissue proteomics. J Proteome Res.

[28] Siddall E, Khatri M, Radhakrishnan J (2017). Capillary leak syndrome: etiologies, pathophysiology, and management. Kidney Int.

[29] Li H, Ortiz R, Tran LT, Salimi-Moosavi H, Malella J, James CA, Lee JW (2013). Simultaneous analysis of multiple monoclonal antibody biotherapeutics by LC-MS/MS method in rat plasma following cassette-dosing. AAPS J.

[30] Peng XY, Liu BN, Li YT, Wang H, Chen X, Guo HZ, Guo QC, Xu J, Wang H, Zhang DP, Dai JX, Hou S, Guo YJ (2015). Development and validation of LC-MS/MS method for the quantitation of infliximab in human serum. Chromatographia.

[31] Law WS, Genin JC, Miess C, Treton G, Warren AP, Lloyd P, Dudal S, Krantz C (2014). Use of generic LC-MS/MS assays to characterize atypical PK profile of a biotherapeutic monoclonal antibody. Bioanalysis.

[32] Heudi O, Barteau S, Zimmer D, Schmidt J, Bill K, Lehmann N, Bauer C, Kretz O (2008). Towards absolute quantification of therapeutic monoclonal antibody in serum by LC-MS/MS using isotope-labeled antibody standard and protein cleavage isotope dilution mass spectrometry. Anal Chem.

[33] Shibata K, Naito T, Okamura J, Hosokawa S, Mineta H, Kawakami J (2017). Simple and rapid LC-MS/MS method for the absolute determination of cetuximab in human serum using an immobilized trypsin. J Pharm Biomed Anal.

